# Translating CO$_2$ variability in a plant growth system into plant dynamics

**DOI:** 10.1038/s41598-022-18058-2

**Published:** 2022-08-15

**Authors:** Tae In Ahn, Je Hyeong Jung, Hyoung Seok Kim, Ju Young Lee

**Affiliations:** Smart Farm Research Center, KIST Gangneung Institute of Natural Products, Gangneung, 25451 Republic of Korea

**Keywords:** Plant sciences, Photosynthesis, Plant physiology

## Abstract

Plant growth occurs owing to the continuous interactions between environmental and genetic factors, and the analysis of plant growth provides crucial information on plant responses. Recent agronomic and analytical methodologies for plant growth require various channels for capturing broader and more dynamic plant traits. In this study, we provide a method of non-invasive growth analyses by translating CO$_2$ variability around a plant. We hypothesized that the cumulative coefficient of variation (CCV) of plant-driven ambient CO$_2$ variation in a plant growth system could yield a numerical indicator that is connected to the plant growth dynamics. Using the system outside-plant growth system-plant coupled dynamic model, we found that the CCV could translate dynamic plant growth under environmental and biophysical constraints. Furthermore, we experimentally demonstrated the application of CCV by using non-airtight growth chamber systems. Our findings may enrich plant growth information channels and assist growers or researchers to analyze plant growth comprehensively.

## Introduction

Measuring and estimating plant growth is routinely employed in agricultural systems and plant research, and non-invasive and high-throughput sensing technologies increase the experimental capacity for capturing plant traits^1^. Such advances in sensor information have yielded novel data and knowledge for developing innovative hypotheses for plant biologists^2^. Thus, enriching channels for capturing plant growth information is an ongoing challenge in advancing plant research and agronomic technologies^1,3^.

In this study, we contribute to non-invasive growth analyses by translating CO$_2$ variability around a plant. Observing and interpreting atmospheric CO$_2$ variability has attracted considerable attention in environmental and ecological context^4^. Some studies observed seasonal variations of CO$_2$ at a regional forest scale and interpreted environmental trends^5^ and ecological vegetation variations^6^. Moreover, recent studies reveal the utility of CO$_2$ variations as data containing rich information, including interactions between plants and the environment. Furthermore, important traits were observed from the magnitude or amplitude of CO$_2$ flux associated with seasonal variations, and the necessity for better characterization and precise measurements was highlighted^4^.

**Figure 1:**
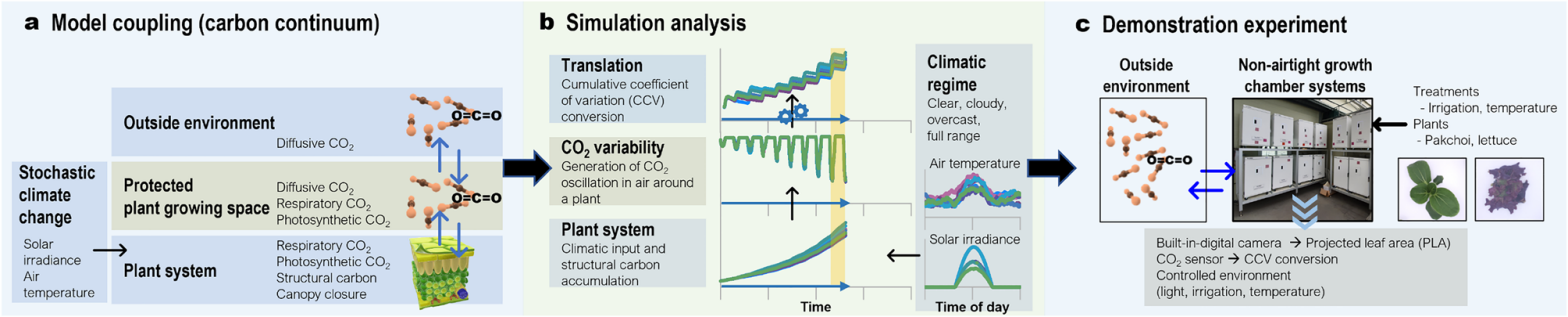
Overall workflow and potential application of CO$_2$ variability translation (**a**) coupled model for CO$_2$ fluxes between the system outside-plant growth system-plant continuum and stochastic climatic inputs. (**b**) Simulation analysis procedure to translate CO$_2$ variability in the plant growth system air into a progress curve displaying environment-plant dynamic interaction and biophysical constraints by cumulative coefficient of variation (CCV) conversion. (**c**) Experimental conditions to demonstrate CCV behaviors under non-airtight growth chamber systems.

**Figure 2:**
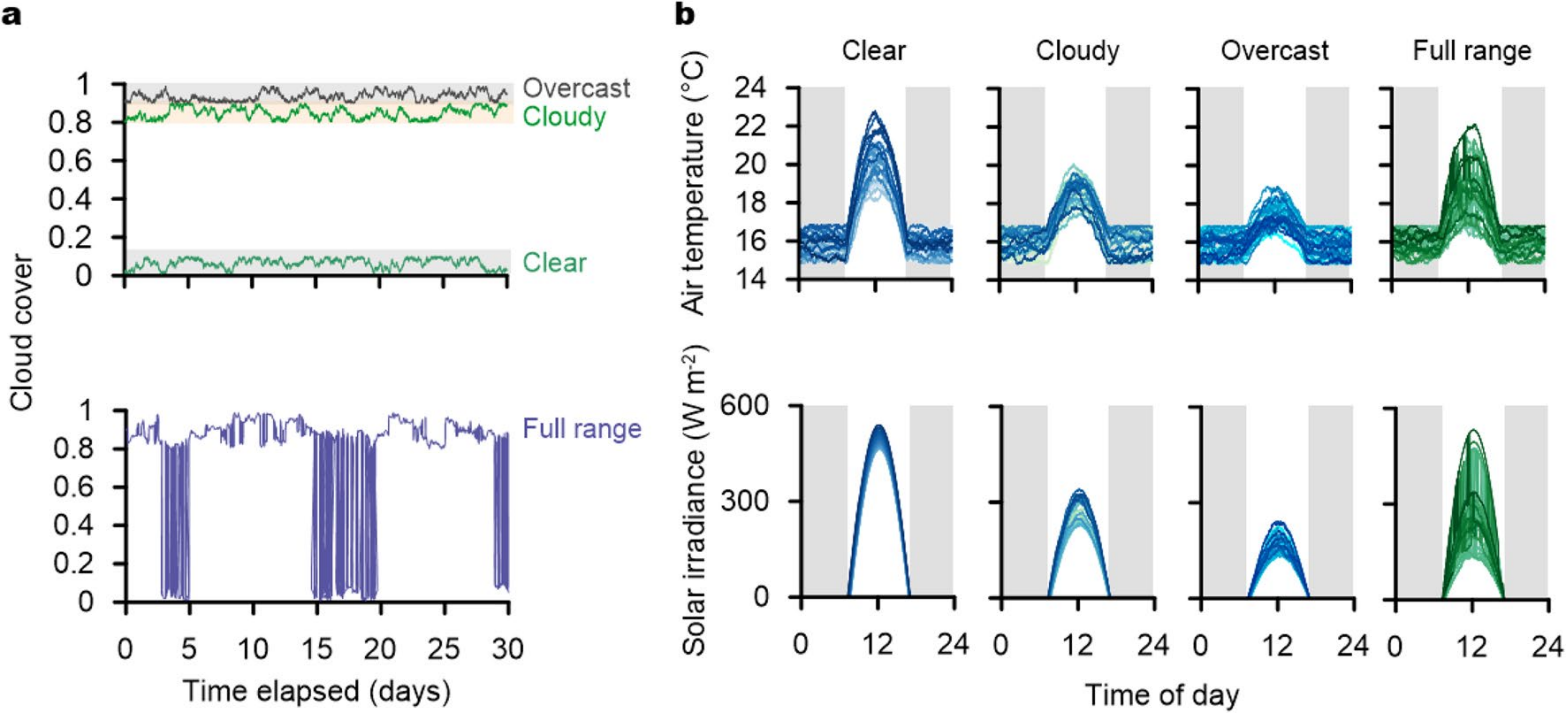
Climatic regimes for the coupled simulation analysis (**a**) representation of the random walk progress for four cloud cover classes and (**b**) superposition of generated curves for the solar irradiance and the air temperature patterns accounting for respective cloud cover classes during all simulation periods.

**Figure 3:**
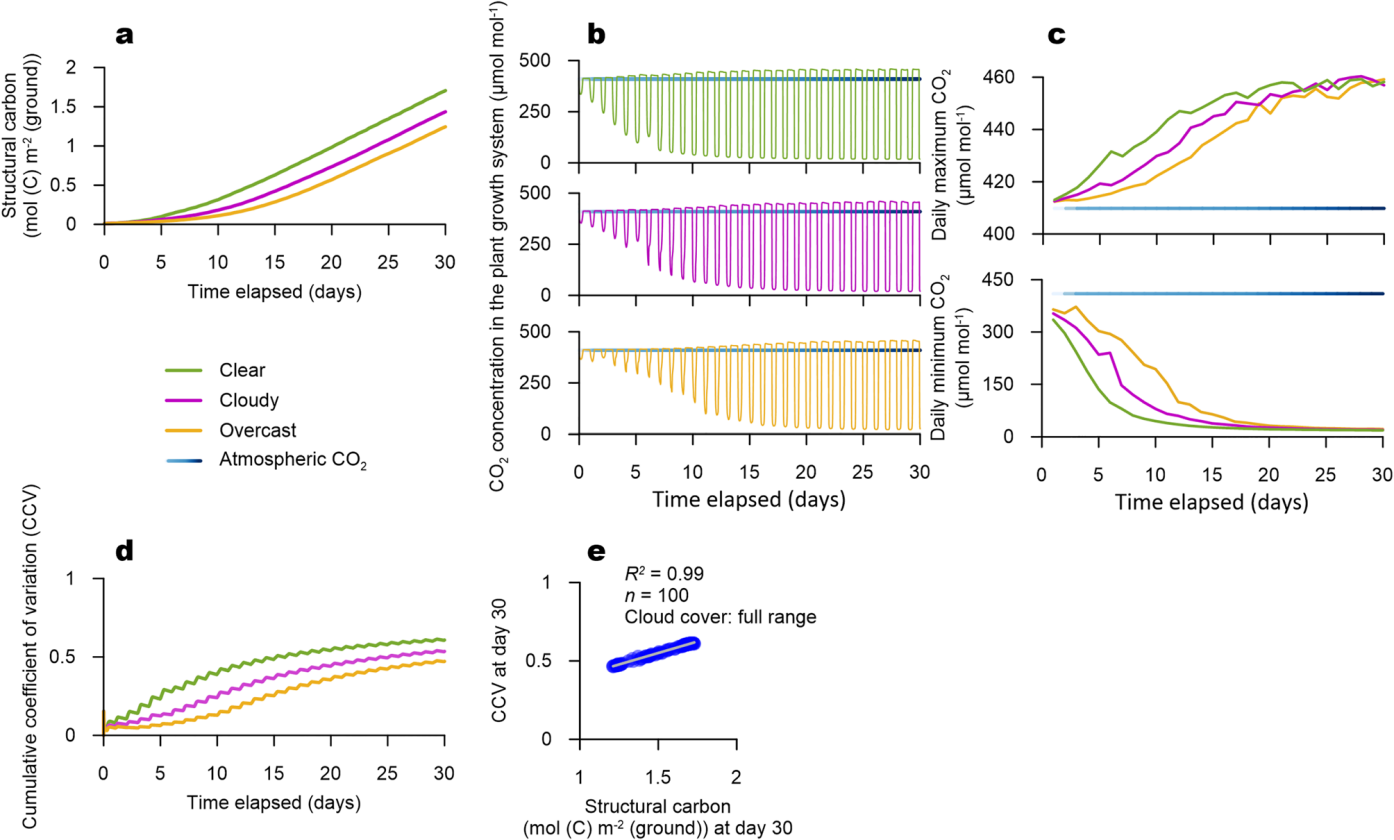
Simulation results of the coupled model Results for structural carbon accumulation in the coupled model simulation (**a**) and corresponding CO$_2$ oscillations in the plant growth system air (**b**). Data filtration of the CO$_2$ oscillations by daily maximum and minimum functions exhibiting gradual deviation of the CO$_2$ variations from the atmospheric CO$_2$ concentration (**c**), CCV progress curves from the three climatic regimes generated sigmoidal curves accounting for each climatic regime (**d**), and the correlation analysis showed a high positive correlation between CCV and structural carbon at day 30 of the full range climatic regime (**e**).

Plant growth systems such as greenhouses and growth chambers contribute to increasing crop productivity and experimental capacities^7,8^. Contrary to the open field, protected cropping conditions such as greenhouses, indoor farms, and growth chambers may limit the impact of mass flow and turbulence on the air exchange between the air around a plant and the atmosphere. Thus, the system may be more favorable in capturing the impacts of plant on the surrounding air, and ambient CO$_{2}$ concentration is one of the representative environmental factors in plant growth systems^9^. However, most of the data generated is still used for monitoring the normal cultivation environment.

CO$_2$ variation data may provide information regarding the contributors of CO$_2$ variability. Plants undergo continuous cycles of photosynthesis and respiration throughout their life, during which CO$_2$ is consumed and emitted via structural growth and maintenance pathways^10^. Once a plant is introduced into an environment, it begins to oscillate the atmospheric CO$_2$^11,12^. We regarded daily CO$_2$ fluctuations as two opposing phases, namely, down- and up-states. The down-state primarily represented the CO$_2$ assimilation rate of a plant, while the up-state was dominated by the CO$_2$ efflux during developmental processes, such as growth, maintenance, and respiration of a plant in C3 and C4 plants. Environmental factors affect physiological processes and plants actively modulate their photosynthetic ability and respiration capacity, accordingly^13^. Contrary to instantaneous photosynthetic rate variables, surrounding CO$_2$ variation is a state variable, which partially mirrors the internal state of the plant. As a result, traces of interactions between the environment and plant traits might be captured in the time-series changes of air around a plant.

**Figure 4:**
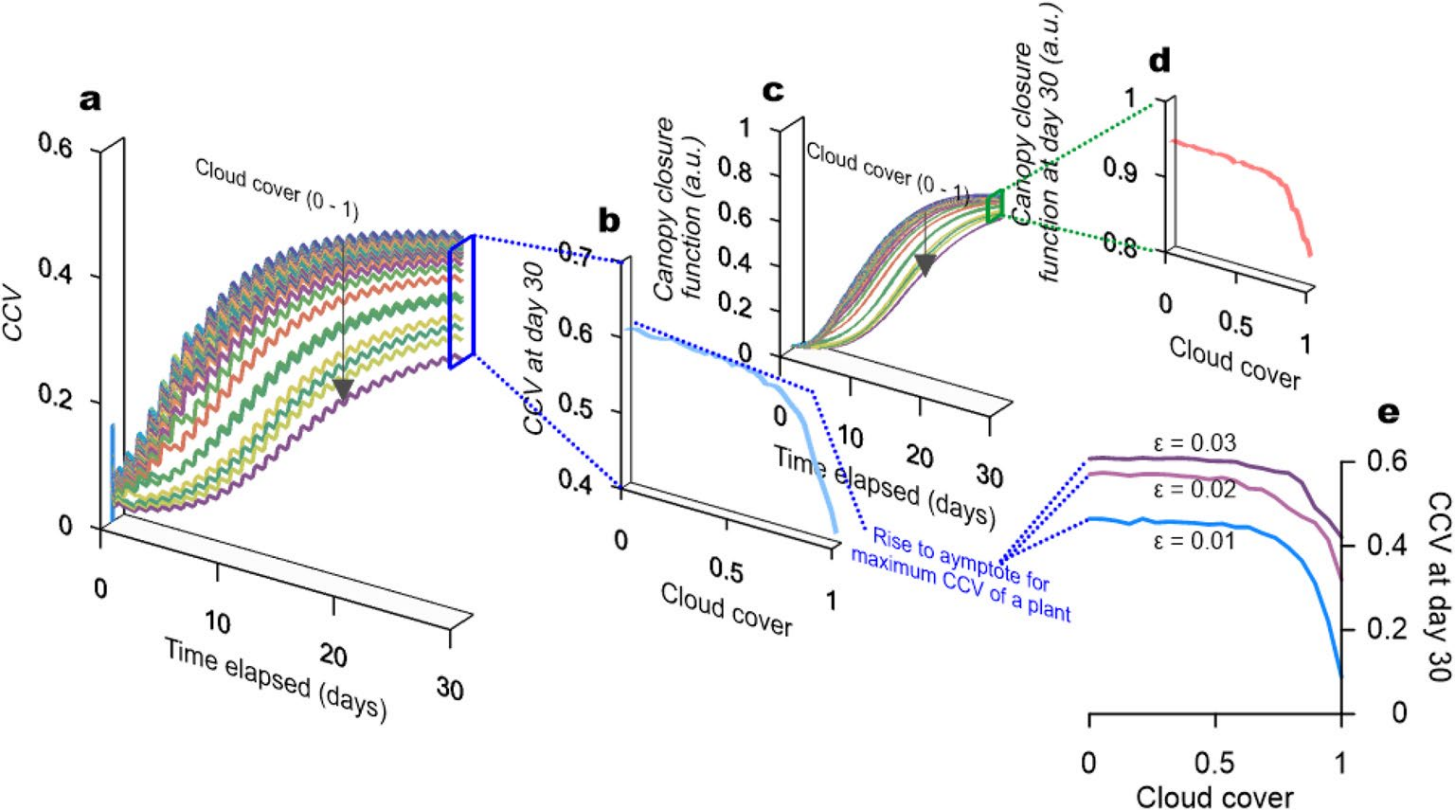
Environment responsive and biophysical characteristics of the CCV curves (**a**) respective CCV progress curves generated by manually increasing the cloud cover value from 0 to 1 and (**b**) cross-sectional graph for CCV versus cloud cover increase at day 30, which shows an asymptotic rise by favorable environmental conditions. Canopy closure progress curve (**c**), cross-sectional graph for the canopy closure versus cloud cover increase at day 30, which is exhibiting an asymptotic rise by favorable environmental conditions (**d**), and distinction between cross-sectional curves for CCV versus cloud cover increases at day 30 according to the parameter manipulating photosynthetic efficiency ($\varepsilon$) (**e**).

**Figure 5:**
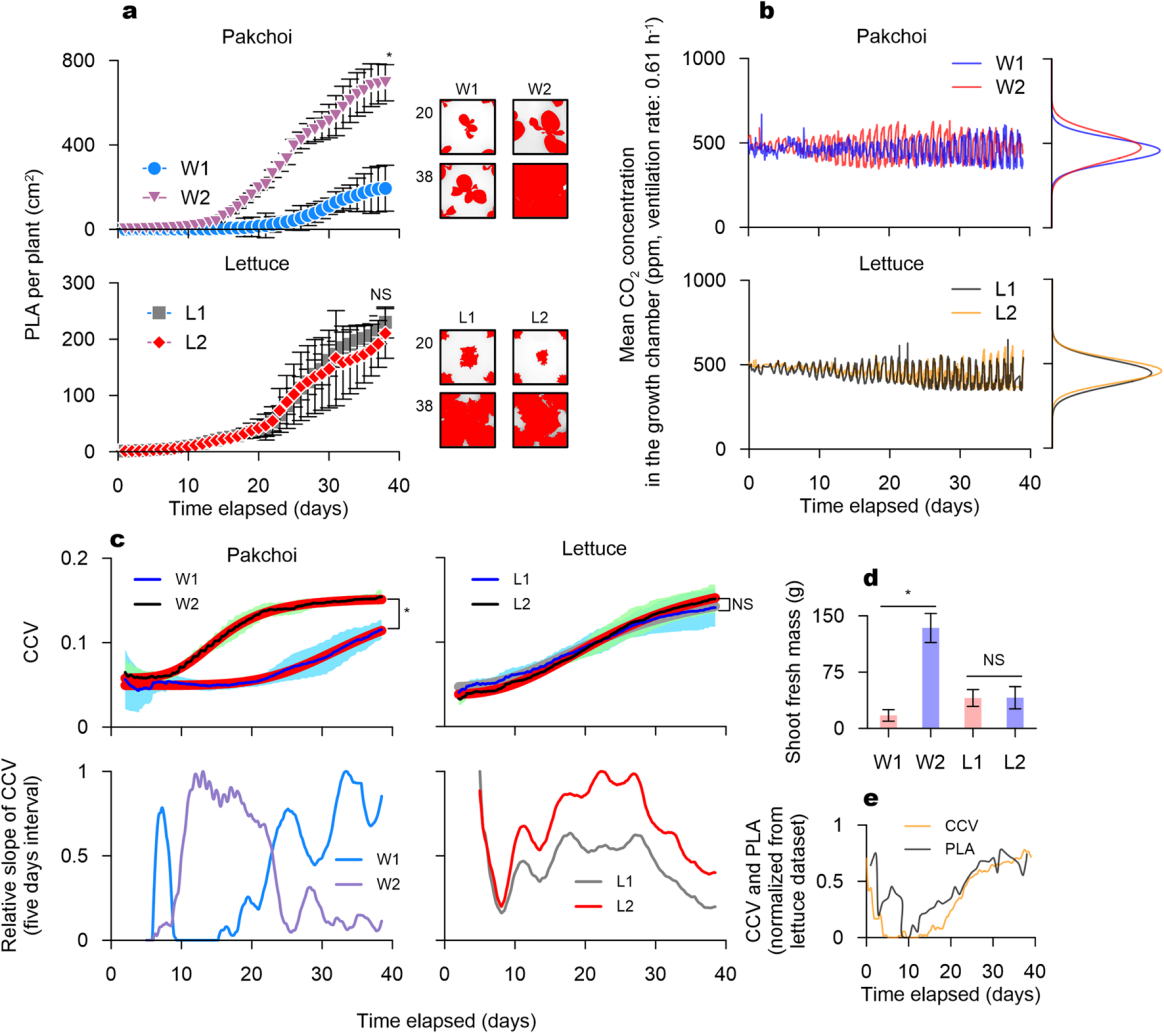
Results of the demonstration experiment Projected leaf area (PLA) progress curves for each treatment (left panels) and representative PLA images segmentation (right panels) for respective growing days (**a**). The data on the final day were statistically compared (*t*-test). NS: not significant (*P* > 0.05); *n* = 3 per treatment. Plant-driven CO$_2$ oscillations according to the treatment (**b**). CCV progress curves and relative slopes (upper panels) of the CCV curves (bottom panels) (**c**). The thin solid lines in the CCV curves indicate the mean of the measured CO$_2$ data from three replicate chambers, shaded areas illustrate the standard deviations of the corresponding mean, and bold solid lines illustrate the sigmoidal fits to the mean curves (R$^{2}$ = 0.988 (W1), 0.998 (W2), 0.998 (L1), and 0.999 (L2)). Comparisons of shoot fresh masses of treatment means (*t*-test) (**d**). NS: not significant (*P* > 0.05); *n* = 15 per treatment. Normalized progress curves of the CCV and PLA (**e**). Normalization function: $x_{nor}=(x-x_{min})/(x_{max}-x_{min})$, where *x* is $x_{nor}$ the normalized value and *x* is the CCV or PLA. $x_{min}$ is the minimum value *x* of CCV or PLA, $x_{max}$ is the maximum value of CCV or PLA at the same time point. Here, *x* corresponds to the lettuce chamber sample. Chambers exhibiting the median level CCV among all lettuce chambers were selected as the sample for *x*.

Therefore, a proper translation of CO$_2$ fluctuations in a plant growth system could provide an opportunity to exploit routine environmental monitoring data as a simultaneous capturing channel for plant traits during experiments and cultivation. As an initial approach to understanding ambient CO$_{2}$ variability, we limited the system boundary to the air in the plant growth system. Furthermore, we only considered a protected plant growth system with diffusive gas exchange between the growth system and the outside environment. We coupled a dynamic model for plant growth (nitrate control in lettuce model, NICOLET model) with the CO$_2$ diffusion process between plant growth system and atmosphere and the daily solar radiation model. We applied a random walk to the total cloud cover to allow the simulation analysis to encompass the various levels of cloud fractions (clear, cloudy, overcast, and full range weather) and its subsequent stochastic results on air temperature, carbon flux, and plant growth. We theoretically investigated CO$_2$ flux between the atmosphere, plant growth system, and the plant and characterized CO$_2$ variability. Although, CO$_2$ fluctuation does not display the intrinsic activity of a plant, the down- and up-state of the diurnal CO$_2$ variations are associated with instant plant activity, and gradual changes in their amplitude may implicate the time-variant physiological capacity of a plant. In this context, the characteristics of these diurnal CO$_2$ behaviors can be obtained by normalizing their variability. Therefore, we hypothesized that the cumulative coefficient of variation (CCV) of the time-series changes of ambient CO$_2$ variation in a plant growth system could yield numerically stable and normalized outputs. Our findings show that the CCV progress curve could translate CO$_2$ variability into plant-environment responsive plant growth information. We experimentally demonstrated CCV applications using non-airtight multiple growth chamber systems and the resulting implications, as an analytic approach, for determining interactions between plant traits and the environment are discussed.

## Methods

### Coupling solar irradiance, temperature, system outside CO$_2$, and CO$_2$ in the plant growth system air using the NICOLET model

The plant growth model was originally developed to simulate dynamic photosynthetic and respiratory carbon flux (growth and maintenance) across vacuoles and plant structures, together with nitrate control in lettuce (NICOLET)^14^. The solar radiation pattern was generated by the total cloud cover variable based on solar elevation changes^15^. A solar irradiation model depicting daily incoming solar radiation at the ground level was used to introduce the diurnal irradiance pattern as an input variable to the NICOLET model. In the solar irradiance model, the incoming solar radiation was modulated by the cloud cover parameter (Supplementary equation (1)). To impose stochastic characteristics on the NICOLET model, and the random walk was applied to the total cloud cover, allowing the simulation analysis to experience various levels of cloud cover. Owing to the close association between air temperature and solar irradiance^16^, an empirical coefficient for correlating the temperature to the daily radiation behaviors for air temperature and solar irradiance was estimated from our weather station data (Supplementary Fig. S1b). However, solely solar radiation is not responsible for air temperature variations, and therefore, the random walk was introduced to the regression slope within the observed variation between the solar irradiance and air temperature (Supplementary Fig. S1c). This was used to generate a stochastic diurnal temperature cycle with reduced dependency on solar irradiance. The random walk applied stochastic daily solar irradiance and temperature patterns coupled to photosynthetic flux modulation (Supplementary equation (12)) and respiratory flux modulation (Supplementary equations (14) and (15)) in the NICOLET model.

The photosynthetic carbon influx and respiratory carbon efflux provided the conjunction point for the system outside-plant growth system-plant continuum mediated by CO$_2$. Therefore, CO$_2$ exchange between system outside, plant growth system, and the plant was coupled (Fig. 1a; Supplementary equation (4)). As an initial step for the CO$_2$ variability utilization, we performed simulation and demonstration experiments in a limited environment. We assumed diffusion as the dominating gas exchange mechanism between the system outside and plant growth system. Thus, non-airtight growth chamber systems without forced ventilation were used in the demonstration experiment. Fick’s Law for molecular diffusion was applied to CO$_2$ exchange within the continuum of system outside-plant growth system-plant. To introduce an identical gas exchangeability between our growth chamber systems and air of the system outside to the simulated ambient air of the plant growth system, the length parameter of the diffusion equation was exploited as a conceptual empirical coefficient by calibrating it to display the best fitted CO$_2$ concentration decay curve to the measured CO$_2$ decay curve in the growth chamber used in this experiment (Supplementary Fig. S2). To determine the CO$_{2}$ concentration of the system outside in the simulation analysis, we referred to the atmospheric CO$_{2}$ concentrations in 2019 (409.8 $\upmu$mol mol$^{-1}$^17^).

### Simulation analysis

Climatic regimes with various combinations of solar irradiance and temperature, within the allocated cloud cover range, were classified as follows: clear, cloudy, overcast, and full range weather (Fig. 1b). Three distinct cloud cover (*N*) ranges were selected: (1) clear ($N \le 0.1$), (2) cloudy ($0.8<N <0.9$), and (3) overcast ($N\ge 0.9$). Cloud cover behavior in the full range class was modulated by another arbitrary variable that was fluctuating between 0 and 1 using random walk behaviors. Therefore, the full-range cloud cover switched between the weather conditions within the three climatic regimes depending on the level of the arbitrary random walk variable. Thus, we exposed the simulated plant to various heterogeneous climatic combinations, and also, manipulated the physiological parameter in the NICOLET model, which modulates the efficiency of photosynthetic carbon flux of a plant ($\varepsilon$; Table S1), to illustrate combined results of physiological and environmental responses.

### Growth chamber experiments

Environmentally controlled growth chamber systems (FarmsCube-20, Korea Digital, Republic of Korea) were used for the demonstration experiments (Fig. 1c). The chambers were equipped with red, blue, and white LED lights, and in the center of the LED panel, a built-in digital camera was installed for a daily photo capture of the plant canopy development. Two leafy vegetables, namely, pakchoi (*Brassica rapa* cv. ‘Green stem’; Asia Seed Co., Republic of Korea) and lettuce (*Lactuca sativa* ‘Danong Yeoreumjeokchukmyeon’; Danong Co., Republic of Korea) were used for the experiments. Nutrient solution was supplied using aeroponics technique, and the solution was supplied into the cultivation container using an irrigation pump (40 $\times$ 40 $\times$ 10 cm) at a 77 mL min$^{-1}$ flow rate per aeroponic nozzle (five nozzles per chamber). The stock nutrient solutions were prepared based on modified Hoagland nutrient solution^18^. Furthermore, the electrical conductivity of nutrient solutions for lettuce and pakchoi chamber experiments were adjusted to 1.2 and 1.9 mS cm$^{-1}$, respectively. Each plant was transplanted into the chamber 14 days after sowing. Two levels of environmental treatments were applied to the pakchoi and lettuce plants. Two irrigation frequencies, (1) 60 s of irrigation every two hours (low irrigation frequency, W1) and (2) 60 s of irrigation every 20 min (high irrigation frequency, W2) were applied for the pakchoi growth chambers, whereas , two temperature variations, (1) 14–21 $^\circ$C (low-temperature range, L1) and (2) 21–25 $^\circ$C (high-temperature range, L2) were applied for lettuce growth chambers. The temperature range of the pakchoi was adjusted to 19–26 $^\circ$C, and the irrigation frequency for the lettuce chambers was 120 s of irrigation every hour. Each growth chamber accommodated five plants, and twelve chambers were used for the demonstration experiment (three chambers for each treatment). The plants were grown for 38 days in the demonstration experiments.

### Projected leaf area (PLA) and cumulative coefficient of variation (CCV) of CO$_2$

Photographs that captured daily images in the growth chambers were used for the estimation of the image-based PLA assessment (Fig. 1c). The cameras took daily photos of the canopy of the growing plants at a pre-programmed time, and the ImageJ software (National Institutes of Health) was used to estimate PLA from the images. The planting plates of each growth chamber had identical widths, and therefore, were used as a measure for the scale sets. Subsequently, all image file types were changed into 8-bit images, and by threshold adjustment, the image was checked against the PLA (cm$^2$) from the background images (Supplementary Fig. S3). The coefficient of variation is defined as the ratio of the standard deviation to the mean^19^:

1 $$\begin{aligned} P_{CV}= \dfrac{s}{\bar{x}} \end{aligned}$$

where $P_{CV}$ is the coefficient of variation, *s* is the standard deviation, and $\bar{x}$ is the mean. The cumulative coefficient of variation was calculated by sequentially increasing the samples to calculate the standard deviation and the mean. In this experiment, CCV calculations were conducted on a time series CO$_2$ dataset. Therefore, the baseline at the sequential increase in the sample numbers corresponded to the initial CO$_2$ data sample, and subsequent data samples were cumulatively introduced for CCV calculation. Moreover, PLA was estimated on a daily basis to experimentally determine whether the CCV conversions yield results were associated with actual plant growth progression.

### Plant material collection and use permission

Permissions were not required for plant material as it was purchased from certified dealer of local area.

### Ethics approval and consent to participate

The study has been conducted without violating any ethical codes of conduct.

## Results

### Generation of weather variation

To determine various life history pathways consisting of heterogeneous climatic combinations of the simulated plant and CO$_2$ of the surrounding air, a random walk process was applied to the cloud cover and the regression slope between solar irradiance and temperature. The three distinct cloud covers (clear, cloudy, and overcast) displayed stochastic behavior within the respective assigned weather levels (Fig. 2a). In contrast, full range cloud cover showed stochastic shifting between the three weather conditions. The 30-day solar irradiance distribution projected on the time of day (24 h) illustrates the distinct solar irradiance pattern between each weather condition during the simulation period (Fig. 2b). Global solar energy is largely, but not solely, responsible for air temperature variations^16^. The random walk process in the regression slope, within the observed variation between the solar irradiance and air temperature in this study, generated a diurnal temperature cycle with reduced dependency on solar irradiance (Supplementary Fig. S2 and Fig. S2b) , while the random walk process in the cloud cover yielded three separate (clear, cloudy, and overcast) and one flexible (full range) stochastic climatic regime on the coupled model.

### Coupled simulation of cloud cover with structural carbon accumulation and CO$_2$ variability

The life history patterns of a plant in the coupled model were consistent with the original study for the NICOLET model^14^ and known trends of a lettuce plant^20^. The structural carbon accumulation patterns of all simulated plants under the distinct climatic regimes, generated by cloud cover manipulation, illustrated initial exponential growth (Fig. 3a). Thereafter, the structural carbon accumulation trends under all three cloud cover ranges shifted to linear growth phases, as derived from canopy closure. Among the three cloud cover ranges, the clear cloud cover condition showed the highest carbon accumulation, followed by cloudy and overcast cloud covers.

CO$_2$ fluctuations in the plant growth system clearly illustrated diurnal patterns resulting from photosynthetic influx and respiratory efflux (Fig. 3b). CO$_2$ in the plant growth system started to oscillate from CO$_2$ concentration of the growing system outside (409.8 $\upmu$mol mol$^{-1}$^17^). All three climatic conditions exhibited distinctive changes in their amplitudes. A gradual but clear decrease in the daily lowest limits in the down-states of CO$_2$ fluctuations was observed under the overcast cloud cover regime, which eventually leveled off after approximately 15 days. The other two cloud cover regimes displayed qualitatively identical results; however, they reached the plateau phase more rapidly. The daily maximum limits in the up-states of all cloud cover ranges also increased from the CO$_2$ level of the growing system outside.

Filtering the simulated CO$_2$ data using the minimum and maximum functions, highlighted the explicit trends of the down- and up-states (Fig. 3c). The daily maximum and minimum CO$_2$ values revealed hyperbolic saturation and decay curves, respectively. In addition, considerable differences were identified between the cloud cover ranges. Notably, the trends highlighted by the data refinement showed sharper details for the different climatic effects on CO$_2$ variability. The daily maximum CO$_2$ concentrations increased over time and were saturated at approximately 450 $\upmu$mol mol$^{-1}$; however, the number of days to reach the maximum plateau differed according to the climatic regime. Clear cloud cover accounted for the most rapid increase in daily maximum CO$_2$, followed by cloudy and overcast conditions. All daily minimum and maximum CO$_2$ concentrations reached saturation after approximately 20 days. The daily minimum CO$_2$ decreased in the following order according to the cloud cover conditions: clear > cloudy > overcast, and the most rapid decline was achieved in the clear climatic regime. The coupled model was able to simulate distinctive trails produced by dynamic carbon fluxes between the plant, air in the plant growth system, and the system outside. These characteristics suggest a basis for a non-invasive connection between plant dynamics and the environment using CO$_2$ variability characterized using CCV.

### Characterization of CO$_2$ variability using the CCV

The raw dataset of the CO$_2$ variations contains diurnal numerical fluctuations (Fig. 3b). Data filtration by daily maximum and minimum values significantly reduces numerical instabilities from the raw dataset, but remains dependent on the unit scale and provides unprocessed information diverged into two respective branches from the down- and up-states. This time-varying periodicity can be further characterized by CCV conversion, as CCV examines the variation in the time-series dataset from a baseline time point. The coefficient of variation normalizes the variation in a dataset, and therefore, the CCV conversion might suggest a comprehensive normalization of these respective traits.

We converted simulated CO$_2$ data into the CCV and found a clear distinction between the different climatic regimes (Fig. 3d). In addition, the CCV on the time series displayed notable pathways accounting for plant developmental progress resulting from climatic differentiation. Under the clear climatic conditions, the CCV exhibited a sharp increase and rapid entry into the stationary phase. A simulated CCV under the cloudy conditions indicated a similar but slightly lowered pattern. In contrast, the overcast condition left a long tail in the pathway to the final simulation day without displaying a specific stationary phase. These results concord with the life history events and characteristics captured by the structural carbon growth curve and daily maximum and minimum value filtration.

Thereafter, we compared the final structural carbon accumulation with the final CCV on the last day of simulation under the full range climatic regime (Fig. 3e). Full range cloud cover manipulation allowed the coupled model to experience various climatic combinations during plant growth. This generated all the climatic conditions across overcast, cloudy, and clear ranges (Fig. 2), and therefore, contrary to the limited climatic variation, the coupled model under the full range cloud cover was able to generate more outputs. The simulation under the full range cloud cover condition generated varying final structural carbon ranging from 1.2 to 1.8 mol (C) m$^{-2}$ (ground). The CCV also showed a wide distribution between 0.42 and 0.61. These results indicate that the CCV accounts for structural carbon accumulation even under varying combinations of solar irradiance and day- and night-time temperatures. The CCV conversion highlights how those traits underneath the ordinary carbon fluctuations in the plant growth system air could be exploited as indicators of plant dynamics.

### Retrieving physiological and species-specific information from the CCV of CO$_2$ variability

The CCV of diurnal CO$_2$ variability was responsive to structural carbon accumulation. In addition, it is noteworthy that the CCV curve consisted of discriminating phases (Fig. 3d). This implies that it is not only characterized by responsiveness, but also by physiological parameters. Therefore, for further characterization, we primarily explored the consequences of the linear variation of cloud cover on the CCV curve geometry.

Increasing the cloud cover parameter identified the relationship between the CCV and cloud cover as a function of time (Fig. 4a). As shown in Fig. 3d, the CCV curves displayed an initial burst phase or a lag phase and then approached a plateau. The cloud cover parameter varied the slopes of the initial phase and the respective levels of the plateau. However, with a decrease in cloud cover, (reaching favorable conditions) the initial slope and level of the CCV saturation point approached a limit. Overall, the CCV curves manifested Hill equation sigmoidal behaviors^21^. The cross-section of the CCV in Fig. 4a on the last day of the simulation along the cloud cover demonstrated another hyperbolic relationship as a function of cloud cover depicting the maximum reachable CCV by a plant species (Fig. 4b). In addition, an identical or nearly identical match in the CCV evolution patterns with canopy structure development (by canopy closure function) was produced concurrently (Fig. 4c, d). This implies that the maximum CCV observed as a function of time (Fig. 4a) or cloud cover (environmental conditions) (Fig. 4b, e) is limited by the biophysical constraints of a plant.

Additionally, we analyzed the consequences of physiological parameter manipulation on the CCV progress curve as a function of cloud cover. We selected one of the physiological parameters in this coupled model, $\varepsilon$, which modulates the efficiency of photosynthetic carbon flux (Supplementary equation (12)) and may represent the intrinsic photosynthetic capacity of a plant species^22^. Three parameters were applied in this analysis ($\varepsilon$ = 0.03, 0.02, and 0.01). The manipulation of $\varepsilon$ yielded changes in the shape of the CCV curves as a function of cloud cover (Fig. 4e). Each level of the maximum reachable CCV on the last day of simulation, dropped in the following order: $\varepsilon$ 0.03 > 0.02 > 0.01.

Therefore, it is theoretically predicted that the CCV of CO$_2$ may generate a curve for plant-environment interactions over time. The CCV progress as a function of time displays sigmoidal behavior, and therefore, each CCV progress curve sequentially represents the exponential, linear, and asymptotic phases over time. Here, their respective asymptotic phases indicate a time point among the life history of a plant species, which is governed by biophysical constraints (Figs. 3d, 4a, c). Furthermore, when the applied environmental conditions satisfy the maximum growth capacity at every time point, the CCV is able to asymptotically reach the maximum limit after a certain time point (Fig. 4b, e). This suggests that CCV may provide information about the potential maximum growth capacity derived from the biophysical limit of an arbitrary plant species. We emphasize that the CCV displays responsiveness to environmental interaction; however, it may also exhibit traits that are numerically stable and convergent to plant-specific biophysical limits.

### Experimental demonstration of the CCV application on CO$_2$ variability

We experimentally demonstrated the translation of plant-environment dynamic interactions by the CCV conversion of CO$_2$ variability. Non-airtight growth chamber systems manipulated environmental conditions while simultaneously collecting CO$_2$ variations, projected leaf area (PLA), and CCV under natural ventilation conditions (ventilation rate: 0.61 h$^{-1}$) (Supplementary Fig. S1). In addition, the final shoot fresh masses were compared. The results of our study reveal a close association between the simulated and measured CCV behaviors (Fig. 5).

We manipulated the water environment of pakchoi and the temperature environment of lettuce growing chambers. Differences in water supply regimes affected the progress of leaf development and led to a clear and significant variations of PLA progression in high (W2) and low (W1) irrigation frequency treatments (Fig. 5a). As determined by the PLA of W2, pakchoi under the W2 treatment exhibited a superior growth performance during the growing period, which generated gradually amplifying evolution patterns in CO$_2$ oscillation (Fig. 5b). The CO$_2$ changes in the W1 treatment also displayed a similar progression; however, the superposed CO$_2$ oscillations of the W1 and W2 treatments showed apparent differences in their amplitude variations. The normalized distribution from CO$_2$ variation in the W1 treatment was centered at approximately 475 $\upmu$mol mol$^{-1}$ with a slightly narrower distribution than that of the W2 treatment (Fig. 5b), whereas the normalized distribution of W2 treatment was centered approximately at 457 $\upmu$mol mol$^{-1}$, a 4% decrease from the W1 treatment.

From these datasets, we found that varying the water environment affected the growth performance of pakchoi and subsequently yielded distinctive diurnal patterns in CO$_2$ oscillations. The CCV conversion of CO$_2$ changes integrated these respective traits as a progress curve over time, and the progress of the CCV curve by the W2 treatment was significantly different from that of the W1 treatment, as was the case with the PLA development (Fig. 5c). Furthermore, the CCV curve from the pakchoi treatments indicated an initial lag phase, a linear phase after short exponential ascending, and a final transition into an asymptotic phase (only in W2 treatment). The average CCV from all W2 chambers remained, and satisfied the components of a typical sigmoidal pattern (R$^{2}$ = 0.99). Throughout all treatments (including L1 and L2), only the W2 treatment, which was abundantly irrigated, exhibited discernible asymptotic behavior in the CCV progression. This progress pattern in the CCV curve concords with the sigmoidal behavior of the CCV curves under cloudy and overcast conditions observed in the simulation analysis (Fig. 4a). The relative slope of the five-day interval CCV outlined the curve transitions observed in the W2 treatment (Fig. 5c). In addition, shoot fresh masses measured at the end of the demonstration experiment confirmed predominant growth in W2 chambers by a significant difference and shoot fresh mass of W1 decreased by 84% compared to that in W2 (Fig. 5d).

The prevailing growth in W2 and also poor growth against unfavorable environments by low irrigation frequency were well depicted by the CCV progress curve (Fig. 5c). For W2, the CCV curve did not plateau during the demonstration experiment. The relative slope of the W2 CCV curve displayed a gradually increasing trend until the end of the experiment (Fig. 5c), suggesting that there could be more room to enhance growth capacity before reaching the full canopy closure, as was simulated in the overcast conditions (Fig. 4).

The datasets acquired from the lettuce growth chambers exhibited nearly identical behaviors to those observed in the pakchoi datasets; however, the different temperature environments in the lettuce chambers did not significantly affect the PLA evolution and the consequent final shoot fresh masses (Fig. 5a, d). The diurnal CO$_2$ patterns of the L2 chambers were indiscernible from L1 patterns and exhibited a similar distribution (Fig. 5b). Furthermore, the relative slope of the CCV exhibited nearly identical behaviors (Fig. 5c). Consequently, the CCV curves acquired from the lettuce chambers were not significantly different from each other (Fig. 5c). Notably, the responsiveness of the CCV curve from a chamber indicates a CCV level comparable to the median value among all the CCVs from the lettuce chambers. The superposed progress of the normalized CCV and PLA curves fluctuated with elapsed time in an almost synchronized fashion (Fig. 5e). The normalization process allocates all ranges of samples into a normalized scale distribution between 0 and 1, which means that for the insignificant sample-to-sample variation, the different signs of progress between the growth capacity of each lettuce chamber were captured by subtle relative differences between the chamber CCVs.

In this demonstration experiment, we did not acquire the theoretically predicted maximum CCV curve pattern using our current environmental treatment. The theoretically predicted maximum CCV curve pattern of a plant species in our simulation displayed a minimized lag phase and progressed close to hyperbolic behavior (Fig. 4a). However, all the CCV curves in this experiment exhibited a near-perfect fit to a sigmoidal curve ($\hbox {R}^{2} > 0.98$) (Fig. 5c), as predicted by the CCV behaviors in the simulation analysis. Furthermore, an extrapolation of the estimated sigmoidal CCV curves of lettuce predicts their asymptote to a higher level (0.171 (L1) and 0.187 (L2)) than that of the CCV of pakchoi (0.149 (W1) and 0.153 (W2)) (Supplementary Fig. S4).

## Discussion

### Carbon-mediated continuum

Our focus on the association of diurnal CO$_2$ variations with the physiological capacity of a plant was built on a carbon-mediated continuum of system outside-plant growth system-plant. Some deviations of gaseous fluxes between the atmosphere and plant growth system allow the air surrounding the plant to be differentiated from the atmospheric air^23^. Previous studies on ambient CO$_2$ variability have shown regional variations in CO$_2$ concentrations. From these data, the authors were able to gain information such as a signal for ecological changes^6^, anthropogenic activities^24^, and climatic impacts^25^. We implemented the situation attainable from flux deviation in the carbon mediated atmospheric continuum where diffusion dominates the gas exchange mechanism between the system outside and the plant growth system. We exploited the length parameter of the diffusion equation to simulate an identical ventilation rate with the non-airtight growth chamber conditions (Supplementary Fig. S2). Therefore, within the CO$_2$ continuum of our coupled simulation, plant growth system air yielded distinct CO$_2$ oscillations from system outside air with gradual evolution trends corresponding to the applied climatic regime (Fig. 3b, c).

### Dynamic responses and biophysical constraints of CCV

Altogether, the simulation analysis led us to theoretically predict three crucial characteristics of the CCV curves, which were (1) the CCV curves respond to the variations in plant growth dynamics due to changes in the ascending slopes of the progress curves (Fig. 3e), (2) the asymptotic rise in the CCV progress curves indicates the maximum reachable biophysical capacity of a plant tailored to the imposed environments (Fig. 3d), and (3) the asymptotic rise in the CCV acquired by imposing favorable climatic conditions predicts the maximum CCV progress curves of a plant species achievable by manipulating the environmental factors (Fig. 4a, b). As predicted in the CCV simulation, we observed the CCV curves displaying an asymptotic phase in the abundantly irrigated treatment of pakchoi (W2). The canopy closure function in the NICOLET model depicts the phenomenon that the light interception capacity for whole plant photosynthesis is limited by the canopy structure of a plant^14,26^. A frequently applied indicator addressing the canopy closure concept is the leaf area index (LAI)^27^. LAI is an important biophysical variable, which is defined as the projected area of leaves per unit ground area. In theory, two distinct species-specific biophysical constraints can be diverged between the LAI and net primary production (NPP) relationship^28^. One is the ceiling LAI type plants exhibiting leveled off behavior in respiration capacity advances beyond a certain LAI. Accordingly, canopy net photosynthesis or NPP also exhibits only a leveled off phase beyond a certain LAI without decline^29^. The other is optimum LAI type plants that display canopy net photosynthesis or NPP decline beyond a certain LAI because of the proportional increase of respiration to the LAI increases^29^. The canopy closure function applied to the NICOLET model supports the ceiling type LAI through its concurrent impact on canopy photosynthesis and respiration (Supplementary equations (10), (11), and (13)). We show that the asymptotic behaviors of the CCV progress curves translate plant internal factors, such as the canopy closure effect and plant growth dynamics. These findings strongly suggest that the CCV progress curve is linked to the inherent LAI characteristics of a plant species to environmental interactions. Although the NICOLET model did not include a canopy closure function for the optimum LAI type plants, we expect that further extension of the model and corresponding experiment would address the progress patterns in the CCV curves. All of the CCV curves from the experiment showed the best fit with the sigmoidal curves (R$^{2}$ = 0.99) (Fig. 5c). In addition, an extrapolation of the sigmoidal model predicted a distinction in the asymptote between the pakchoi and lettuce plants (Supplementary Fig. S4). The lettuce and pakchoi that we used have structurally distinctive three-dimensional canopy shapes and individual leaves. The combinatory effects of the environmental and biophysical interactions are challenging to study, and some studies have raised concerns regarding the contrasting results derived from the combination of individual leaf photosynthesis and canopy structure^30,31^. This study did not progress the cultivation period until the actual asymptote. However, we note the potential of the predicted asymptote as another species-specific discriminator.

Here, we demonstrated how the CCV conversion of plant-driven ambient CO$_2$ oscillations could be exploited for plant growth dynamics within the life-history scale. CCV curve illustrates the time series interaction between the environment and the biophysical constraints of a plant species. In the current study, we did not experimentally observe the asymptotic approaches for the maximum CCV curve reachable by the most favorable environmental conditions. However, there may be possibilities for finding convergent trends of a maximum CCV progress curve as a spatiotemporally ideal baseline for the growth of a plant species by further experimental trials to seek optimal environmental combinations. Furthermore, the demonstration experiment of the temperature treatment for the lettuce did not result in significant differences in the CCV curve, PLA, and final shoot fresh mass. We applied two different temperature ranges (L1: 14-21; L2: 21–25) to the lettuce chambers; however, under indoor cultivation conditions, these correspond to optimal or sub-optimal environmental conditions for the lettuce growth^32^, and thus may not have acted as a sufficient constraint to draw a significant difference.

As an initial approach to exploit ambient CO$_2$ variability, a limited experimental condition was applied, and we observed CCV patterns generation associating plant traits under this condition. Thus, under the extended condition where intermittent mass flow and air turbulence are actively involved in the gas exchange process between the atmosphere and a plant growth system may limit CCV translation. Additionally, we analyzed the impact of plant internal parameters based on the NICOLET model in this study. Thus, there may be partial limitations for mechanistically matching parametric behaviors of the model to the CCV behaviors which derived from the overall variations inside the plant. However, further refinement of units, scaling relationships, standardization, signal processing, and analysis relating the variation inside the plant may help develop robustness and applicability of the translation processes.

Recent plant growth systems accumulate various environmental data, and CO$_2$ is one such representative environmental factor that is collected by these systems. However, most plant growth systems routinely generate CO$_2$ sensing data for monitoring purposes. Consequently, our findings provide an entry point to utilize CO$_2$ sensing data monitored in growth systems, which may assist in enhancing the ability to interpret crop growth performance or cultivation experiment results.

## Supplementary Information

Supplementary Information.

## Data Availability

The datasets generated during and/or analysed during the current study are available from the corresponding author on reasonable request.
