# Supplementary material for: Translating CO2 variability in a plant growth system into plant dynamics
Source: Sci Rep. 2022 Aug 15;12:13809. doi: 10.1038/s41598-022-18058-2 (PMC9378742; doi:10.1038/s41598-022-18058-2)
Supplement: Supplementary file 1 — Supplementary Information. [file 41598_2022_18058_MOESM1_ESM.pdf]

Supplementary Information

Translating CO_2 variability in a plant growth system into plant dynamics

Tae In Ahn, Je Hyeong Jung, Hyoung Seok Kim, Ju Young Lee*

Smart Farm Research Center, KIST Gangneung Institute of Natural Products, Gangneung 25451, Republic of Korea

* Corresponding author

Tel: +82-33-650-3703; Fax: +82-33-650-3629; E-mail address: jyl7318@kist.re.kr

Coupled model detail

Solar irradiance

The solar radiation pattern was generated by the total cloud cover variable based on solar elevation changes [1]:

$$K^{+} = K_0^{+}(1 + b_1 N^{b_2}) \quad \text{(S1)}$$

where K^{+} is the reduced solar radiation by the total cloud cover (W m^{-2}); K_0^{+} is the incoming solar radiation at ground level under clear skies (W m^{-2}), which is determined by solar elevation over seasonal time changes; b_1 and b_2 are the empirical coefficients; and N is the total cloud cover. N is a value between 0 and 1; closer to 0 corresponds to a clear day, and closer to 1 corresponds to a cloudy day. K_0^{+} was estimated following the numerical procedure of Holtslag and Van Ulden [1]. The unit for light intensity (I) used in the coupled NICOLET model was photosynthetically active photon (PAP) per unit ground area per unit time (mol (PAP) m^{-2} (ground) min^{-1}). Here, mol (PAP) m^{-2} (ground) min^{-1} conversion from W m^{-2} for the NICOLET model input was obtained by multiplying K^{+} (W m^{-2}) by the conversion constant of sun and skylight source (4.57 μ mol m^{-2} s^{-1} per W m^{-2}) [2]. More specifically, one watt per square meter is equal to 1 J m^{-2} s^{-1} and one micromole is equal to 10^{-6} mole. Overall, the solar irradiance as an input variable of the coupled NICOLET model (I) was obtained by converting time and molar units of the converted K^{+} (K^{+} multiplied by the conversion constant of sun and skylight source).

Temperature (an empirical model for air temperature)

We established a simple linear regression model between air temperature and solar irradiance based on close correspondence between solar irradiance and air temperature.

Subsequently, we introduced a random walk process to the regression slope within the observed daily slope variation range. Solar irradiance (W m^{-2}) and air temperature (°C) were collected from the weather station installed on the KIST Gangneung Institute of Natural Products (37.8°N, 128.8°E) from June to August 2021. We converted the weather station data to PAP per unit ground area per unit time (mol (PAP) m^{-2} (ground) min^{-1}; I in the NICOLET model) for solar irradiance and Kelvin temperature scale (K; T_a in the NICOLET model) for air temperature, respectively (Fig. S1a, e). During the data collection period, the regression slope and intercept for solar irradiance and the air temperature was 31.80 and 294.5, respectively (R = 0.61, $P < 0.001$; Fig. S1b). We estimated daily slope and intercept variation within the period of weather station data collection by using the SLOPE and INTERCEPT Excel function. Next, a random walk process was introduced to the regression slope and intercept within the range of the daily slope and intercept variation. For each time step, a random number between -5 and 5 with a unit increment of 0.5 was added to the slope and the intercept of the previous time step. The random walk slope and intercept changes for each data interval (hour) were guided to drift between the 5th and 95th percentile normal distribution of the slope variation and the 5th and 70th percentile normal distribution of the intercept variation, respectively (Fig. S1c, d).

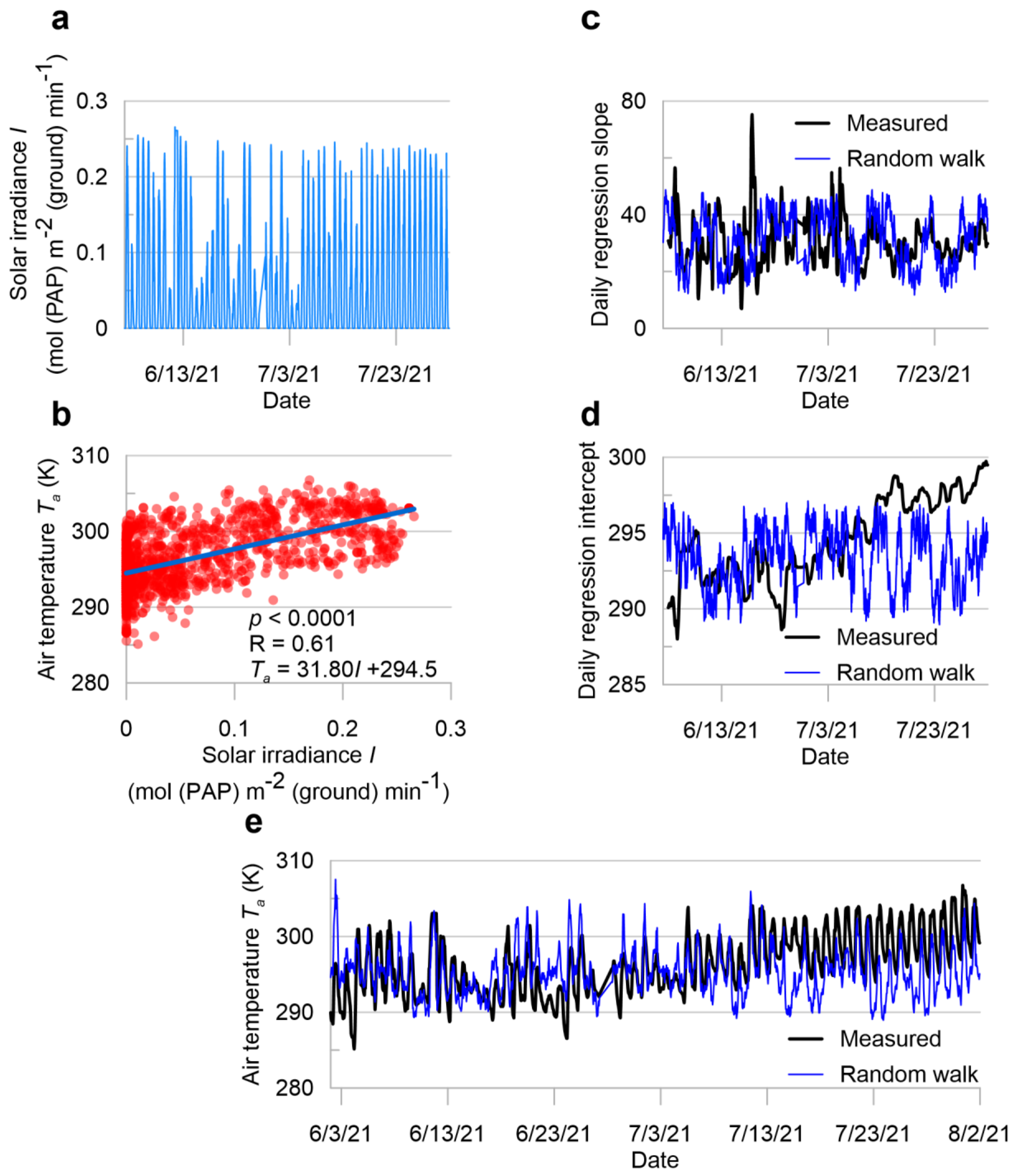

Fig. S1 a random walk applied empirical model for air temperature

(a) Solar irradiance (I) changes during the data collection period; the solar irradiance in the original data from the weather station was converted from W m^{-2} to mol (PAP) m^{-2} (ground) min^{-1}. (b) Regression model for solar irradiance and air temperature data. (c) Daily regression

slope changes of concurrent changes in air temperature and solar irradiance during the data collection period; the thick black solid line indicates measured data, and the thin blue solid line indicates random walk applied data. (d) Daily regression intercept changes of concurrent changes in air temperature and solar irradiance during the data collection period; the thick black solid line indicates measured data, and the thin blue solid line indicates random walk applied data. (e) Air temperature (T_a) changes during the data collection period; the thick black solid line indicates measured data, and the thin blue solid line indicates random walk applied data.

System outside CO_2 and CO_2 in the plant growth system (Diffusion model fitting for the simulation of CO_2 in the plant growth system)

We established a carbon-mediated continuum of system outside-plant growth system-plant. Diffusional flow contribution in the coupled model continuum was expressed by Fick's Law for molecular diffusion:

$$F_{Cd} = D_C(C_{Ca} - C_{Cg})/Z \quad \text{(S2)}$$

where F_{Cd} is the diffusional flow (mol (C) m^{-2} min^{-1}); D_C the diffusion coefficient of CO_2 (m^2 min^{-1}); C_{Ca} is the atmospheric CO_2 concentration (mol (C) m^{-3}); C_{Cg} is the CO_2 concentration in the plant growth system; and Z is the length of the diffusion region, and here in our study, we exploited the parameter as a conceptual, empirical coefficient for calibrating the simulated CO_2 concentration decay curve to the measured CO_2 decay curve in the growth chamber used in this experiment.

The decay method was used for the estimation of the ventilation rate of the growth chamber and calibration of the length parameter (Z) in the diffusional flow equation (Supplementary equation (2)). The decay method can be used when a space is vacated, and the

ventilation rate can be estimated using measured CO_2 concentrations over the decay period. We used exhalation to differentiate growth chamber CO_2 concentration from the atmospheric CO_2 concentration (Fig. S2). Under the condition of increased CO_2 from the atmosphere, the ventilation rate of the growth chamber could be estimated using two CO_2 measurements [3]:

$$A_D = 1/\Delta t \ \ln\{(C_1 - C_R)/(C_0 - C_R)\} \qquad \text{(S3)}$$

where A_D is the ventilation rate (h^{-1}) Δt is period between measurements (h); C_0 and C_1 is measured CO_2 concentration in the growth chamber over the decay period; C_R is CO_2 concentration in the steady-state concentration, which is equivalent to ambient atmospheric concentration.

The length parameter (Z) in the diffusional flow (F_{Cd}; Supplementary equation (2)) in the coupled model continuum was calibrated to the measured decay curve from the growth chamber. The carbon balances of a carbon-mediated continuum of system outside-plant growth system-plant are:

$$\frac{dC_{Cg}}{dt} = F_{Cd} - F_{Cp} + F_{Cg} + F_{Cm} \qquad \text{(S4)}$$

where F_{Cp} (mol (C) m^{-2} min^{-1}) is photosynthetic carbon influx into a plant system; F_{Cg} (mol (C) m^{-2} min^{-1}) and F_{Cm} (mol (C) m^{-2} min^{-1}) are growth respiration and maintenance respiration carbon effluxes, respectively. C_R in Supplementary equation (3) correspond to C_{Ca} in Supplementary equations (2) and (4). We substituted C_R for C_{Ca} and 0 for F_{Cp}, F_{Cg}, and F_{Cm}, respectively, in Supplementary equations (2) and (4) during the calibration process. However, the concentration unit in the NICOLET model is molar concentration-basis. Thus, we converted measured PPM (parts per million by volume in air) to molar concentration in air (mol (C) m^{-3}) and vice versa. Next, the length parameter (Z) Supplementary equation (2) and

(4) was calibrated by a progress curve analysis that estimated the value that minimized the root mean square error (RMSE) between the measured and simulated values of the growth chamber CO_2 decay curves (Fig. S2).

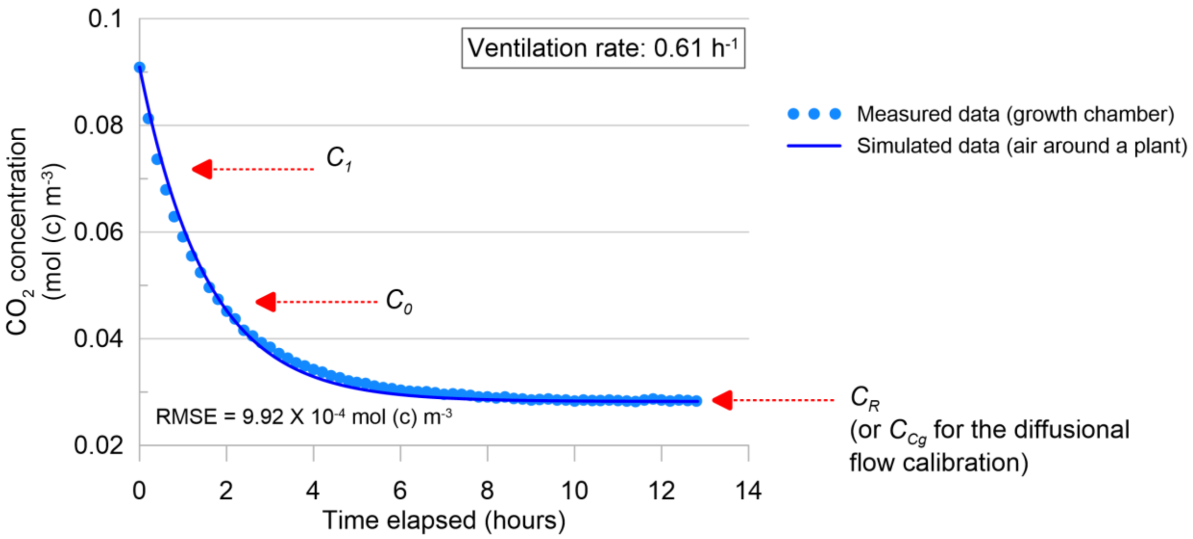

Fig. S2 Estimation of the ventilation rate of the growth chamber from the measured data and the length parameter calibration in the diffusional flow equation (Supplementary equation (2)).

NICOLET model

We coupled the NICOLET model with solar irradiance, temperature, atmospheric CO_2, and CO_2 in the plant growth system to address plant growth and CO_2 variability under comprehensive interactions between different key components of the plant environment. NICOLET model was designed to predict the behavior of carbon flux in the structure and non-structural carbon and was validated for lettuce growth simulation [4]. Also, the NICOLET model can simulate structural carbon accumulation of a plant under both nitrogen-stressed and

nitrogen-abundant conditions. In our study, we applied the nitrogen-abundant condition to the coupled NICOLET model. Introducing an atmospheric CO_2 diffusion and daily solar radiation model enabled the interpretation of CO_2 variability around a plant together with the dynamic plant carbon accumulation and released under variations in diurnal environmental patterns.

The carbon balances of the NICOLET model are:

$$\frac{dM_{Cv}}{dt} = F_{Cp} - F_{Cvs} - F_{Cg} - F_{Cm} - F_{Cve} + F_{Cev} \quad \text{(S5)}$$

$$\frac{dM_{Cs}}{dt} = F_{Cvs} \quad \text{(S6)}$$

$$\frac{dM_{Ce}}{dt} = F_{Cve} - F_{Cev} \quad \text{(S7)}$$

where M_C (mol (C) m^{-2}) is the molar mass of carbon per unit ground area and the subscript v, s, and e refer to the vacuole, structure, and excess-C compartments in a plant system, respectively; and F_c (mol (C) m^{-2} min^{-1}) is the molar flux of carbon in a plant system and the subscript *vs*, *ve*, and *ev* refer to the fluxes 'from the vacuole to the structure', 'from the vacuole to the excess-C compartments', and 'from the excess-C compartments to the vacuole', respectively.

Crucial carbon fluxes for our coupled model continuum are F_{Cp}, F_{Cvs}, F_{Cm}, and F_{Cg} and these are formulated as:

$$F_{Cp} = p\{I, C_{Cg}\} f_s\{M_{Cs}\} A_p\{\gamma_{Cv}\} \quad \text{(S8)}$$

$$F_{Cvs} = g\{T_a\} f_s\{M_{Cs}\} A_{Vs}\{\gamma_{Cv}\} \quad \text{(S9)}$$

$$F_{Cm} = e\{T_a\} f_s\{M_{Cs}\} \quad \text{(S10)}$$

$$F_{Cg} = \theta F_{Cvs} \quad \text{(S11)}$$

where p, f_s, A_p (or A_{Vs}), and e (or g) refer to the unattenuated gross photosynthesis of a

closed canopy, a fraction of canopy light interception, attenuation factor governed by carbon-flow limitations, and the exponential dependence of respiration (maintenance and growth) on temperature, respectively; and θ is a constant fraction for growth respiratory flux.

These fluxes are functions of the light intensity (I), CO_2 concentration in the plant growth system (C_{Cg}), and of the state of a plant, via M_{Cs} (molar mass of the structural carbon of a plant) and γ_{Cv} (normalized vacuolar carbon concentration). For proper interpretation of CO_2 variation in the plant growth system, we focused on p, f_s, e, and g factors in the above flux equations and these are formulated, respectively as:

$$p\{I, C_{Cg}\} = \frac{\varepsilon I \sigma C_{Cg}}{\varepsilon I + \sigma C_{Cg}} \quad \text{(S12)}$$

$$f_s\{M_{Cs}\} = 1 - \exp\{-aM_{Cs}\} \quad \text{(S13)}$$

$$e\{T_a\} = k \exp\{c(T_a - T*)\} \quad \text{(S14)}$$

$$g\{T_a\} = ve\{T_a\} \quad \text{(S15)}$$

where ε, σ, a, k, c and v are the constant coefficient of the NICOLET model; T_a and $T*$ are air temperature and an arbitrary reference temperature, respectively. Supplementary equation (13) approaches 1 asymptotically, and this equation is frequently used for the attenuation of canopy photosynthesis rate by canopy light interception [5]. Supplementary equation (14) modulates temperature dependence of respiration (maintenance and growth). However, temperature dependency above the optimum level was not considered in the NICOLET model [4]. Thus, the temperature dependency of respiration in the NICOLET model is not valid for high temperatures. The rest of the details in the NICOLET model equations and parameters was conducted following the numerical procedure of Seginer[4]. The parameters values are listed in Table S1.

PLA assessment by ImageJ software

The ImageJ software (National Institutes of Health) was used to estimate PLA from the top view images of the growing plants in the growth chambers. We imported the image files into ImageJ and width of the top plate of the cultivation bed was applied as a scale for each image files (Fig. S3a). Next, we converted the scale set image into an 8-bit image (Fig. S3b) and adjusted the color threshold to highlight pixels of the projected leaf area (PLA) in the image (Fig. S3c). Next, we used the Rectangular Selection tool in the ImageJ toolbar to specify the plant-covered area and selected Area and Limit to Threshold checkboxes in the Set Measurement preference (Fig. S3c). Finally, we selected Measure tool to assess the PLA (Fig. S3d).

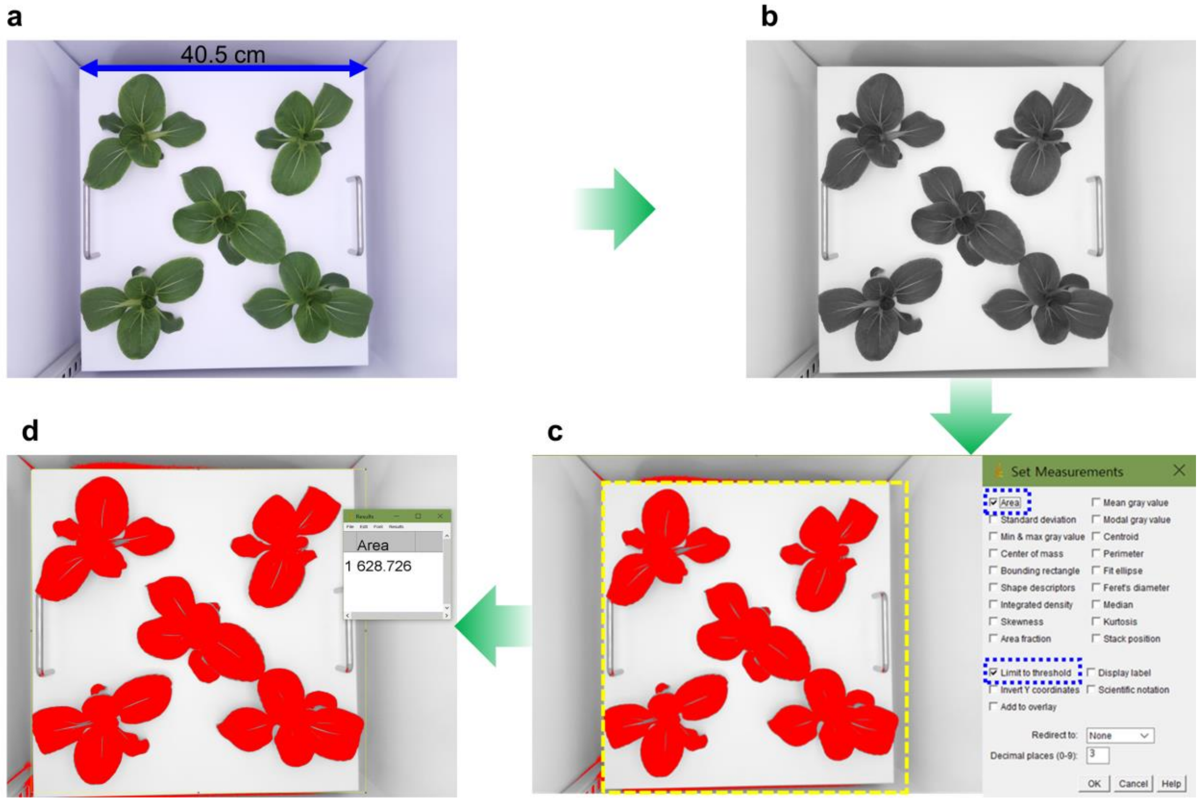

Fig. S3 PLA assessment procedure using ImageJ software

(a) Original image and scale setting of the image file. (b) 8-bit image conversion. (c) Selection of the plant covered area and Set Measurement preference (d) PLA assessment.

Extrapolation of the estimated sigmoidal CCV curves

We extrapolated the estimated sigmoidal CCV curves in Fig. 5c. The sigmoidal CCV curves for each treatment were progressed as a function of time (days) until the asymptotes were explicitly observed (Fig. S4).

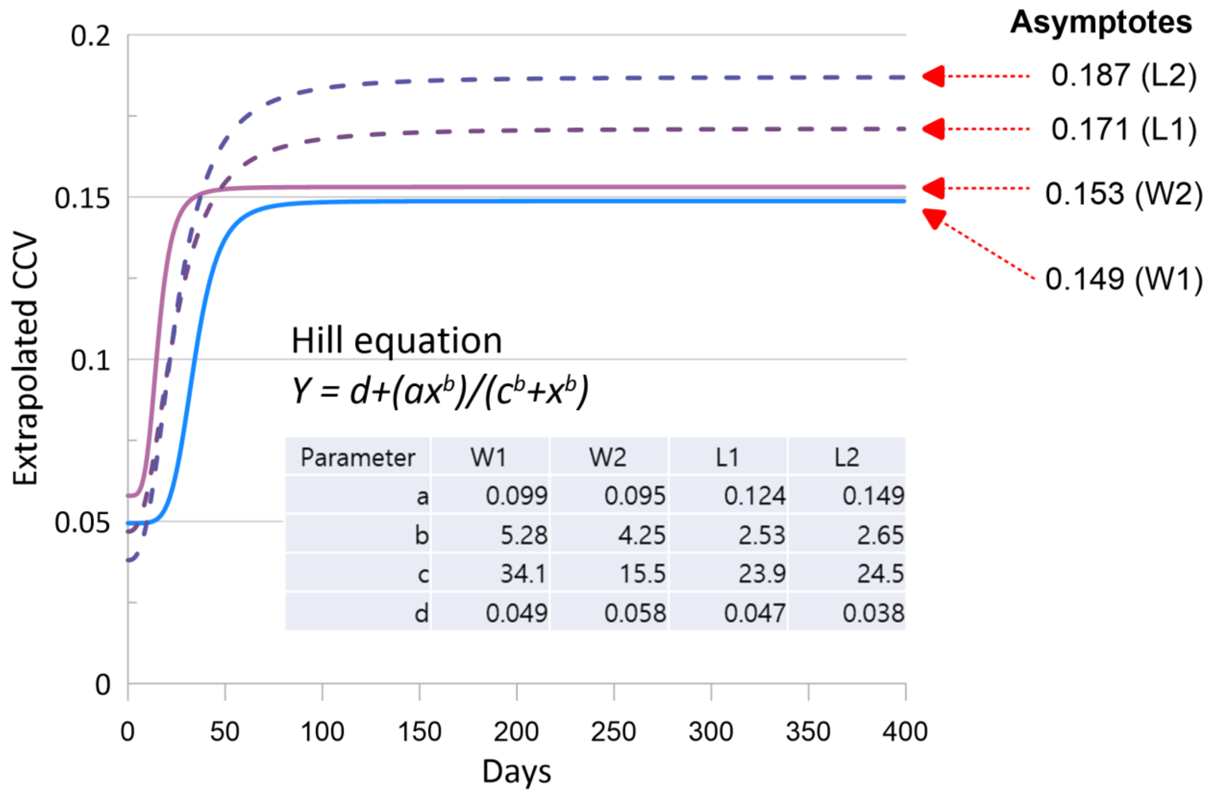

Fig. S4 Extrapolation of the estimated CCV curves in Fig. 5c

Table S1. Parameter values

Parameter	Definition	Value	Unit	Source
$\boldsymbol{b_1}$	Empirical coefficient (solar radiation)	-0.75		Holtslag and Van Ulden [1]
$\boldsymbol{b_2}$	Empirical coefficient (solar radiation)	3.4		Holtslag and Van Ulden [1]
$\boldsymbol{D_C}$	Diffusion coefficient of CO_2	9.6×10^{-4}	m^2 min^{-1}	
$\boldsymbol{C_{Ca}}$	Atmospheric CO_2 concentration	0.0172 (409.8 ppm)	mol (C) m^{-3}	
$\boldsymbol{Z}$	Length of the diffusion region	0.0886	m	
$\boldsymbol{A_D}$	Ventilation rate	0.61	h^{-1}	Estimated from the growth chamber
$\boldsymbol{\varepsilon}$	Photosynthetic efficiency	0.03 (for the simulation without parameter manipulation)	mol (C) mol^{-1} (PAP)	Seginer [4]
$\boldsymbol{\sigma}$	Leaf conductance to CO_2	6.0×10^{-3}	m s^{-1}	Seginer [4]
$\boldsymbol{a}$	Light extinction coefficient	1.7	m^2 (ground) mol^{-1} (C)	Seginer [4]
$\boldsymbol{k}$	Contant coefficient for respiration	0.25×10^{-6}	mol (c) m^{-2} (ground) s^{-1}	Seginer [4]
$\boldsymbol{v}$	Contant coefficient for respiration	9.5		Seginer [4]
$\boldsymbol{\theta}$	Constant fraction for growth respiratory flux	0.3		Seginer [4]

Reference

1. Holtslag, A. A. M. & Van Ulden, A. P. A simple scheme for daytime estimates of the surface fluxes from routine weather data. *J. Climate Appl. Meteor.* **22**, 517–529 (1983).
2. Thimijan, R. & Heins, R. Photometric, radiometric, and quantum light units of measure: a review of procedures for interconversion. *HortScience* **18**, 818–822 (1983).
3. Batterman, S. Review and Extension of CO2-Based Methods to Determine Ventilation Rates with Application to School Classrooms. *Int J Environ Res Public Health* **14**, 145 (2017).
4. Seginer, I. A dynamic model for nitrogen-stressed lettuce. *Ann. Bot.* **91**, 623–635 (2003).
5. Thornley, J. H. M. & Johnson, I. R. *Plant and Crop Modelling: A Mathematical Approach to Plant and Crop Physiology*. (Oxford University Press, 1990).
